# Editorial: Advancing measurement validity in clinical and dynamic psychology

**DOI:** 10.3389/fpsyg.2026.1924253

**Published:** 2026-10-02

**Authors:** Anna Panzeri, Adam Klocek, Stefania Mannarini, Alessandro Alberto Rossi

**Affiliations:** 1 Department of Human and Social Sciences, University of Bergamo, Bergamo, Italy; 2 Psychology Research Institute (INPSY), Faculty of Social Studies, Masaryk University, Brno, Czechia; 3 Institute of Psychology, Czech Academy of Sciences (PSU CAS), Brno, Czechia; 4 Centre of Social and Psychological Sciences, Slovak Academy of Sciences, Košice, Slovakia; 5 Department of Philosophy, Sociology, Education, and Applied Psychology, Section of Applied Psychology, University of Padova, Padova, Italy; 6 Department of Philosophy, Sociology, Education, and Applied Psychology, Section of Applied Psychology, Center for Intervention and Research on Family Studies (CIRF), University of Padova, Padova, Italy

**Keywords:** clinical and dynamic psychology, clinimetrics, construct validation, measurement, patient-reported outcomes, psychometrics, questionable measurement practices, validity

Clinical and dynamic psychology involve objects that cannot be held in the hand. Psychosis risk, anhedonia, dysregulated rage, embodied cognition, and spiritual wellbeing—these are latent constructs inferred from a person's responses rather than read off a dial. A cardiologist can point to a stenosed artery; we can only point to a score and argue about its meaning. That asymmetry carries consequences that the field too easily forgets: in clinical and dynamic psychology, the instrument is the evidence—or, more precisely, the inference we draw from its score is and that inference holds only when a validity argument ties the observed response to a clearly specified population, context, and purpose (Cronbach and Meehl, 1955; Messick, 1995). When the instrument is miscalibrated, every inference built on it inherits the error: diagnoses misclassify, genuine change goes undetected, and interventions are aimed at something that was never quite there.

This Research Topic takes this idea and puts it to work. Its contributions span new scale development, cross-cultural adaptation, re-examination of established screening tools, and item-level psychometric analysis. They all share the stance that gives this editorial its title. Validity is not a certificate that a test earns once and keeps. Rather, it is a cumulative, revisable body of evidence that must be re-earned whenever the measure moves to a new population, language, setting, decision, or moment in a patient's course. Properly understood, validation never ends. We argue that this never-ending quality is not a nuisance to be wished away but the very form that scientific and clinical responsibility takes when our objects are latent.

## 1 Validity is never finished

Outside the clinic, the case for measurement rigor is by now familiar. Validity is a unitary, evolving argument about whether scores support the inferences we draw from them, not a property that a test simply possesses (Messick, 1995). The replication crisis is, in large part, a measurement crisis: noisy, poorly validated instruments do not merely shrink effects—they can inflate and distort them under selection for statistical significance (Loken and Gelman, 2017). A striking share of psychology's measurement failures trace not to exotic statistics but to a “measurement schmeasurement” attitude, in which scoring and validation decisions go unreported, unexamined, and improvised on the fly (Flake and Fried, 2020; Flake et al., 2017, 2022). No sample size and no analytic sophistication can repair an invalid measure.

In clinical and dynamic psychology, the stakes differ in kind, because the person who bears the cost of a bad measurement is not a reader but a patient. The clinimetric tradition emphasizes this point: patient-reported outcomes earn their place through interpretability, responsiveness, and usefulness for the individual case, not through internal homogeneity alone (Balsamo et al., 2019; Carrozzino et al., 2021; Panzeri et al., 2026; Rossi et al., 2024a,b). Therefore, what a clinically grounded appraisal asks of any instrument is narrow and demanding at once: not whether it is tidy, but whether it helps the clinician understand the person and decide what to do next (Balsamo et al., 2019).

## 2 What the contributions teach

The studies gathered here do not argue this thesis in the abstract; they enact it, each taking responsibility for an instrument at a different stage of its life. Read for their clinical payload rather than their statistics, they fall into three categories.

### 2.1 Naming what patients feel

Some clinical experiences have outrun the tools meant to capture them. Michałkiewicz et al. give dysregulated anger a careful anatomy, separating its affective and behavioral facets in the Anatomy of Rage Scale; Wiesmann et al. operationalize the intrusive “high-place” urge—the call of the void—that clinicians often hear described but rarely measure; and Xu et al. refine the assessment of anhedonia with a Specific Loss of Interest and Pleasure Scale that retains its meaning from community samples through to patients. What unites these studies is their discipline at the front end: the construct is defined by theory and lived experience before any number is attached to it (Clark and Watson, 1995). Dickens et al. make the stakes explicit, showing how laypersons actually read the items of the Authentic and Hubristic Pride Scales—because when a respondent and clinician do not share the same understanding of a word, the resulting score measures confusion rather than the patient's state (Gehlbach and Brinkworth, 2011; Flake and Fried, 2020). A high reliability coefficient cannot rescue a measure whose items are read in divergent ways, and asking one narrow question in many ways only flatters the statistic while starving the clinical picture (Sijtsma, 2009; Nunnally and Bernstein, 1994).

### 2.2 Questioning the frontline screen

Other contributions turn a skeptical eye on instruments already in daily use, where complacency is most dangerous. Sergi et al. reevaluate the factor structure of the Montreal Cognitive Assessment, and D'Ignazio et al. ask whether the Mini-Mental State Examination can truly separate mild cognitive impairment from ordinary aging; neither study treats a familiar screening tool as beyond question. Tolomeo et al. apply Item Response Theory to the Sniffin' Sticks identification subtest, showing how precision in detecting olfactory loss of neurological significance depends on the behavior of individual items. Balhara et al. anchor a screen for problematic internet and device use to an explicit ICD-11 definition rather than to convenience. The shared warning is the one that Fried's work made unavoidable: a single total score can hide wholly different patients behind an identical number; therefore, what appears to be one disorder may in fact be many (Fried and Nesse, 2015; Fried, 2015, 2017; Bottesi et al., 2015). What clinical work requires is a screen that grades severity and tracks change, not one that merely correlates (Carrozzino et al., 2021).

### 2.3 The same score for different people

Finally, several studies confront the quiet assumption that a measure has the same meaning for everyone who completes it. Barajas et al. validate a Spanish CAARMS-S so that psychosis-risk interviews retain their meaning beyond their language of origin; Süto et al. bring a multidimensional inventory of religious and spiritual wellbeing into Hungarian; and Zheng et al. build an embodied-cognition scale for Chinese university students—each treating cultural adaptation as fresh validation rather than mere translation. Kristensen and Vestad emphasize this point, cautioning that some apparent robust sex differences in adolescent mental health may be artifacts of non-equivalent measurement rather than facts about young people. Where comparability fails, the same cut-off silently misclassifies different groups in different ways, and a difference manufactured by the instrument is mistaken for a difference in the patients. Establishing measurement invariance is therefore a precondition for any group comparison, not an optional refinement (Putnick and Bornstein, 2016).

## 3 A manifesto for never-ending, patient-centered validation

Taken together, these contributions converge on a single mandate: a measure earns trust only by proving itself where it will actually be used—not on a convenient student sample, displayed thereafter the same way as a trophy. For clinical and dynamic psychology, we distill that mandate into four commitments to never-ending validation (Table 1).

1. *Define before you count*. Specify the construct from theory and lived experience and confirm that respondents read it as intended before any number is attached.
2. *Distrust the familiar screen*. Treat widespread use as a reason for scrutiny, not a substitute for it; one total score may conceal distinct patients behind a single number.
3. *Establish equivalence before comparing*. Adaptation demands new evidence regarding item meaning, response options, structure, and interpretation in the target context—translation is not validation.
4. *Measure change, not just consistency*. A clinical instrument must move with the patient, remain tolerable to someone who is unwell, and fit the realities of day-to-day clinical practice (Fitzpatrick et al., 1998; Mokkink et al., 2010).

**Table 1:** Four commitments of validation: a practical standard for measurement validity in clinical and dynamic psychology.

No.	Commitment	What it requires	Risk if ignored
1	Define before you count	Specify the construct from theory and lived experience, and confirm respondents read items as intended - before any number is attached.	The score measures confusion, not the patient; no reliability coefficient can repair that.
2	Distrust the familiar screen	Treat widespread use as a reason for scrutiny, not a substitute for it: re-examine structure, cut-offs, and item functioning.	One total score hides distinct patients behind a single number - one disorder/construct may be many.
3	Establish equivalence before comparing	Before comparing groups, cultures, or sexes, show the measure means the same thing in each. Translation is not validation.	The same cut-off misclassifies different groups, and a difference made by the instrument is mistaken for a real one.
4	Measure change, not just consistency	A clinical instrument must move with the patient, stay tolerable when they are unwell, and fit the working day of real services.	The instrument is deaf to the very change that clinical decisions depend on.

None of the four is met once and for all; each is renewed as patients deteriorate and recover, as measures cross borders, and as the decisions a score must support keep changing.

This Research Topic collectively enacts these four commitments; each pairs a principle with the clinical failure it prevents, and none is satisfied once and for all.

None of these commitments is met once and for all. Each must be renewed as patients deteriorate and recover, as measures cross borders, and as the decisions we ask a score to support keep changing. That is what never-ending validation means: the day we declare a clinical measure finally, finished, and universally “valid” is the day we stop watching it, and stop protecting the individuals it exists to serve. In clinical and dynamic psychology, validation that never ends is not a burden but the precise shape that care takes when the thing we are measuring is the human mind.
